# Service delay in schizophrenia: case–control study of pathways to care among homeless and non-homeless patients

**DOI:** 10.1192/bjo.2025.19

**Published:** 2025-03-25

**Authors:** Ida-Marie Mølstrøm, Rasmus Handest, Mads Gram Henriksen, Annick Urfer Parnas, Julie Nordgaard

**Affiliations:** Mental Health Center Amager, Capital Region Psychiatry, Copenhagen, Denmark; Psychiatry East, Region Zealand, Roskilde, Denmark; Centre for Subjectivity Research, Department of Communication, University of Copenhagen, Copenhagen, Denmark; Department of Clinical Medicine, University of Copenhagen, Copenhagen, Denmark

**Keywords:** Psychosis, prognosis, homelessness, social psychiatry, course of illness

## Abstract

**Background:**

Early detection of psychosis is paramount for reducing the duration of untreated psychosis (DUP). One key factor contributing to extended DUP is service delay – the time from initial contact with psychiatric services to diagnosis. Reducing service delay depends on prompt identification of psychosis. Patients with schizophrenia and severe social impairment have been found to have prolonged DUP. Whether service delay significantly contributes to prolonged DUP in this group is unclear.

**Aim:**

To examine and compare the course of illness for patients with schizophrenia who are homeless or domiciled, with a focus on service delay in detecting psychosis.

**Method:**

In this case–control study, we included out-patients with a schizophrenia spectrum diagnosis and who were homeless or domiciled but in need of an outreach team to secure continuous treatment. Interviews included psychosocial history and psychopathological and social functioning scales.

**Results:**

We included 85 patients with schizophrenia spectrum disorder. Mean service delay was significantly longer in the homeless group (5.5 years) compared with the domiciled group (2.5 years, *P* = 0.001), with a total sample mean of 3.9 years. Similarly, DUP was significantly longer in the homeless group, mean 15.5 years, versus 5.0 years in the domiciled group (*P* < 0.001). Furthermore, the homeless group had an earlier onset of illness than the domiciled group but were almost the same age at diagnosis.

**Conclusions:**

Our findings point to the concerning circumstance that individuals with considerable risk of developing severe schizophrenia experience a substantial delay in diagnosis and do not receive timely treatment.

Schizophrenia affects approximately 1–2% of the world’s population^1^ and is considered to be among the most severe psychiatric disorders. The onset of this disorder usually occurs in early adulthood and can lead to long-term impairments and significant difficulties for patients and their relatives. Outcomes vary significantly, ranging from full recovery to more or less chronic symptoms and impaired social functioning.^2,3^ Moreover, individuals with schizophrenia are generally at risk of stigma, discrimination, social isolation, unemployment and loneliness.^4,5^

The years immediately following the onset of psychosis have been coined as a window of opportunity for intervention. Duration of untreated psychosis (DUP) has consistently been found to be correlated to factors related to illness progression, such as treatment response and outcome.^6,7^ The cause of this relationship remains unclear.^8^ Nonetheless, a shorter DUP implies early treatment following the onset of psychosis, thus reducing the duration of suffering from an untreated mental illness and decreasing the risks of adverse consequences such as substance use and suicidal behaviour.^9^

In the past 20 years there has been a focus on the critical period of the early course of schizophrenia, aiming to improve detection and intervention for patients with first-episode psychosis. Randomised clinical trials have demonstrated that early specialised interventions positively affect short-term outcomes, such as reducing symptom severity and psychiatric hospitalisations and improving involvement in school or work.^10^

Naturally, early-intervention programmes rely heavily on the diagnostic assessment that leads to inclusion in such programmes. Timely diagnosis is of paramount importance, and this hinges on the recognition of psychotic symptoms. Nonetheless, studies have found significant delays between entering the mental healthcare system and receiving the correct diagnosis and treatment.^11,12^ Delay in diagnosis, also called service delay, is a form of diagnostic error, which is defined as a missed, wrong or delayed diagnosis^13^ that may lead to inappropriate treatment and postponement of appropriate help such as antipsychotic medication or psychosocial and psychotherapeutic interventions.^13,14^ Such service delay can also contribute to premature discharge from hospitals or out-patient care services. Changing diagnoses may also undermine the patient’s trust in mental health services and increase the risk of stigmatisation associated with suffering from multiple mental disorders. It can also lead to a loss of hope in recovery, which can harm empowerment and recovery.^15,16^

## Aim

In this study, we retrospectively examined and compared the course of illness for patients with a schizophrenia spectrum diagnosis (SSD), who were either homeless or did have a home but required outreach psychiatric assistance, with a special focus on delays in detecting psychosis.

## Method

We conducted an explorative case–control study in Copenhagen, Denmark. We adhered to the Strengthening the Reporting of Observational Studies in Epidemiology guidelines to ensure our observational study’s transparent and comprehensive reporting.^17^

### Participants and setting

The study was conducted at Mental Health Centre Amager, a general psychiatric hospital in Copenhagen. Patients from two outreach psychiatric teams were included: one treating homeless patients with suspected psychotic disorders and another for patients with severe psychiatric disorders. All participants were evaluated by their psychiatrists to ensure that they were eligible and capable of participating. This evaluation was reassessed during all consecutive interviews. Participants received written and verbal information about the study, and those meeting the inclusion criteria were invited to participate. All participants gave written informed consent before participation.

Eligible patients were patients with a confirmed or suspected diagnosis of SSD (including schizophrenia, schizotypal disorder and schizoaffective disorder).

The authors assert that this work complies with the ethical standards of the relevant national and institutional committees on human experimentation, and with the Helsinki Declaration of 1975 as revised in 2013. The National Committee on Health Research Ethics in Denmark exempts interview studies from medical ethical review owing to their non-invasive nature.

### Sample 1

Sample 1 included 35 patients who were in contact with the Homeless Outreach Psychiatric Service (HOPS). HOPS was initiated in 2012 by the Municipality of Copenhagen in collaboration with the Mental Health Services in the Capital Region of Denmark. HOPS seeks out homeless individuals with psychosis in Copenhagen and offers them psychiatric evaluation and treatment.^18^ Inclusion criteria were homelessness for at least 1 month and a confirmed or suspected diagnosis of SSD. Exclusion criteria were forensic status and inability to participate in conversations for at least 30 min. Patients were recruited over a period of 20 months; the final tranche of patients was included in August 2023. The clinical staff at the HOPS team consecutively referred potentially eligible patients to the research project, and author R.H. then met with the patient at the shelter, clinic or on the street for an initial meeting. If eligible, and following written and verbal consent, the interviews were planned as involving between four and eight sessions and additional cognitive tests. Author R.H., a medical doctor with extensive psychiatric and research experience, conducted the interviews, which were video or audio recorded and discussed with authors J.N. and M.G.H.

### Sample 2

Patients were recruited from a psychiatric out-patient clinic for severe mental illness in Copenhagen. This clinic is organised as flexible assertive community treatment (FACT) teams. Patients were recruited over a period of 23 months, the final patient being recruited in October 2023. The clinical staff of the FACT teams consecutively referred potentially eligible patients, who were then contacted by telephone and invited for an initial meeting about the project. If eligible, and following written and verbal consent, the interviews were planned as between two and four sessions with additional cognitive tests. Eligible patients were those who met the following criteria:

(a) a confirmed or suspected diagnosis of SSD (diagnosed or confirmed by psychiatrists in the out-patient clinic in accordance with The International Classification of Diseases, 10th revision (ICD-10));
(b) impaired social functioning, as defined by (i) unemployment and (ii) in need of outreach services (unable to meet regularly for appointments in the clinic, resulting in domiciliary visits, being assisted for appointments in the clinic and periods with sporadic contact or missed appointments);
(c) living independently, i.e. not in sheltered housing or being homeless, in a psychiatric residential facility, or being homeless.

Exclusion criteria were forensic status and an inability to engage in conversations for at least 30 min.

Author I.-M.M. conducted all interviews, which were video or audio recorded and subsequently discussed with J.N. and M.G.H. Interviews were conducted at either the out-patient clinic or the patients’ homes, accommodating their preferences.

### Clinical assessment

All patients underwent a comprehensive psychopathological evaluation, including detailed psychosocial history and course of illness interview, which was further elaborated upon by medical records.

The psychopathological examination included the operational criteria checklist for psychotic illness and affective illness (OPCRIT),^19^ parts of the schedule for affective disorders and schizophrenia,^20^ the positive and negative syndrome scale (PANSS),^21^ the examination of anomalous self-experience (EASE)^22^ and the global assessment of functioning (GAF).^23^ The patients received a research diagnosis according to ICD-10.^24^

IQ was assessed using the computer-assisted test Intelligence Structure Test 2000 R, assessing verbal–, numerical– and figurative–spatial IQ by three selected subtests: analogies, sequences of numbers and matrices. We then summarised the results from those subtests into a global IQ score.^25^ If patients were unable to complete the entire test battery, assessments were conducted using a smaller test battery, including PANSS and GAF. All interviews were conducted in Danish unless the participant preferred English, and the checklist and scales have all been validated in Danish. Authors I.-M.M. and R.H. were reliability trained for the assessments used.

### Definitions

The onset of illness was defined as the earliest age at which the patient had sought medical or other treatment – e.g. a primary care physician or a psychologist – for any psychiatric symptom(s) or when the psychiatric symptoms had started to severely affect social functioning. The onset of psychosis was defined as the onset of any significantly present psychotic symptom. For some participants, the time of the first psychotic symptoms could not be established or recalled and, in these cases, we noted the onset of psychosis as the first description of psychotic symptoms in the medical records.

Duration of untreated psychosis was defined as the time between the onset of psychosis to receiving either relevant treatment (e.g. antipsychotic medication) or a diagnosis of schizophrenia or other non-affective psychosis (i.e. ICD-10 F20-29, except F21). Similarly, duration of untreated illness (DUI) was defined as the time from the onset of illness until either receiving relevant treatment or a diagnosis of schizophrenia or other non-affective psychosis. Help-seeking delay was defined as the time from the first psychotic symptom to initial contact with psychiatric services.^26^ Service delay was defined as the timespan between first contact with professional mental health services to receiving either adequate treatment or a diagnosis of schizophrenia or other non-affective psychosis. Participants diagnosed with schizotypal disorder were not included in these analyses.

A series of interviews were consensus rated, with both raters trained and tested according to consensus ratings. Furthermore, the group met for monthly co-rating meetings. Inter-rater reliability was assessed using Cohen’s kappa. The agreement between I.-M.M. and consensus was 0.93, while that between R.H. and consensus was 0.94. Average kappa score was 0.94, indicating a high level of agreement. Inter-rater reliability for the onset of psychosis for I.-M.M. and R.H. was assessed using the intraclass correlation coefficient (ICC, two-way random effects, absolute agreement); this calculation is recommended for continuous variables. The calculated ICC was 0.95, indicating a high level of agreement between raters.

### Post hoc assessment

From the interviews, we noted a tendency among homeless patients to display signs of hostility and opposition. Crucially, this was not directed particularly towards the interviewer but in their everyday lives and as a general attitude towards others and the world. By contrast, domiciled patients tended to exhibit signs of overcompliance in both the interview setting and their everyday lives. The attitudes of opposition and overcompliance have been described in schizophrenia by Binswanger.^27^ Binswanger reconsidered Bleuler’s concept of autism in schizophrenia, arguing that it is not a state of extreme self-sufficiency but of strong dependence on others, resulting from a disturbed relation to the social world, and which may manifest in attitudes of opposition or overcompliance.^28^

Based on this impression, which emerged throughout data collection, we selected and classified symptoms and signs from our psychopathological examination into two groups: opposition *v*. overcompliance. This was done before any statistical tests were carried out. We aimed to compare the scores of opposition and overcompliance between domiciled and homeless patients to determine whether our impression was also reflected in the data.

I.-M.M. initially chose symptoms and signs, with consensus about their relevance and allocation to each group obtained by I.-M.M., M.G.H. and J.N. We chose symptoms and signs that we thought could reflect the descriptions of opposition and overcompliance described by Binswanger^27–29^, and partly by Catalano and Green^30^, i.e. symptoms and signs that we thought could be expressive of a disturbed relation to the social world (e.g. social anxiety or guilt feelings could indicate a tendency toward overcompliance, whereas hostility and paranoid anxiety could indicate a tendency toward opposition).

### Statistical analysis

Statistical analysis was conducted using R 4.3.1^31^ and IBM SPSS Statistics 29.0.1.0.^32^

For categorical variables, we report both the number and percentage, and potential differences between the two groups were examined by chi-squared tests. Numerical values were tested for normal distribution, and mean, median and standard deviations are reported. We applied Bonferroni correction to account for multiple comparisons and reduce the risk of type I error. For each analysis where multiple comparisons were made, the threshold significant *P*-level (0.05) was divided by the number of comparisons within that set. Pearson’s product–moment correlation was conducted to examine the relationship between IQ and GAF scores.

We performed multiple linear regression analysis examining correlations between service delay, help-seeking delay, DUI and DUP, and the variable group (homeless or domiciled), as well as multiple potential confounders: age at non-affective psychosis diagnosis, sex, substance use, education level, migrant status, location of first contact (psychiatric emergency room, child and adolescent psychiatry, out-patient clinic, private practice), previous diagnosis of anxiety or affective disorder, previous diagnosis of neurodevelopmental disorder and previous diagnosis of schizotypal disorder. These confounders were chosen after examining the existing literature on factors influencing pathways to care.^11,12^ For service delay, we also included help-seeking delay in the analysis. Patients with a diagnosis of schizotypal disorder were excluded from calculations of DUP, DUI and help-seeking and service delay. DAGitty v.3.1 was used to create a directed acyclic graph (DAG).^33^

## Results

We included 85 patients in the study: 35 from the homeless outreach team (homeless group) and 50 from the psychiatric out-patient clinic for severe mental illness (domiciled group).

Both groups had mean GAF <50 on both symptoms and functioning scales, which would be categorised as severe illness.^23^ IQ was significantly associated with GAF symptom score (*r* = 0.42, *P* = 0.001) but not with GAF disability score (*r* = 0.07, *P* = 0.6). Overall information on basic demographics for both groups is given in Table 1.

**Table 1:** Sample characteristics

	Homeless group	Domiciled group	Overall	Statistics
Variable	*n* = 35	*n* = 50	*N* = 85	*P*-value
Gender (female)	6 (17%)	26 (52%)	32 (38%)	*0.001*
Age (mean (s.d.))	32.6 (11.2)	43.7 (12.3)	39.1 (13.0)	*<0.001*
Never married or cohabiting	19 (54%)	28 (56%)	47 (55%)	0.9
Has children	9 (26%)	10 (20%)	19 (22%)	0.5
Social isolation	28 (80%)	42 (84%)	70 (83%)	0.6
Citizenship (Danish)	29 (83%)	42 (84%)	71 (84%)	0.9
GAF symptom (mean (s.d.))	32.2 (7.2)	38.4 (7.2)	35.9 (7.8)	*<0.001*
GAF functioning (mean (s.d.))	25.0 (6.1)	31.1 (5.1)	28.6 (6.3)	*<0.001*
IQ total score (mean (s.d.))	92 (16)	93 (15)	93 (15)	0.8
Suicidal thoughts, current	26 (74%)	12 (24%)	38 (45%)	*<0.001*
Suicide attempts	14 (40%)	20 (40%)	34 (40%)	>0.9
Number of interviews (mean (s.d.))	5.3 (1.5)	3.7 (1.4)	4.3 (1.7)	*<0.001*
Missed interview appointments (mean (s.d.))	3.5 (3.8)	1.0 (1.6)	2.0 (3.0)	*<0.001*
Education				
Ten years or less	19 (54%)	23 (47%)	42 (50%)	0.5
High school or equivalent	11 (31%)	17 (35%)	28 (33%)	0.8
Higher education	1 (3%)	3 (6%)	4 (5%)	0.6
Started but did not complete university	4 (11%)	1 (2%)	5 (6%)	>0.9
University, completed	0 (0%)	5 (10%)	5 (6%)	0.06

IQ, Intelligence Structure Test 2000 R; GAF, global assessment of functioning.

Bonferroni correction was applied for multiple comparisons. The adjusted significance level is *α* = 0.003 (0.05/18.0). Significant *P*-values are reported in italics.

### Homeless group

The homeless group consisted of 29 males and 6 females. Interviews were conducted at the shelters, in the out-patient clinic or on the street, and patients were frequently reminded of the appointments via telephone or by the staff in their shelters if they had consented to this. Nonetheless, we had a high rate of no-shows (approximately 40% of appointments). In 25 of the 35 patients, the contact was disturbed by psychotic symptoms or formal thought disorders.

### Domiciled group

We included 50 patients from the psychiatric out-patient clinic with severe mental illness: 26 females and 24 males. Forty-two lived alone, three lived with a partner, four lived with family and one lived with a roommate. All were unemployed, and 25 were receiving early retirement benefits. Ten had children but none were living with their children. All had difficulties meeting regularly for appointments in the out-patient clinic. Twenty-two had all visits from the psychiatric clinic at home, due either to anxiety or the severity of their psychotic illness, 3 had some of their meetings at home, 19 had extensive periods of missed appointments and no-shows and 6 were assisted in getting to appointments by family members or others.

Interviews were conducted with patients at their homes, the out-patient clinic or the psychiatric in-patient unit and were arranged based on their availability and preferences; 28% of the interviews resulted in cancellations or no-shows. On average, 3.7 interviews were conducted to complete the entire interview process.

### Diagnosis and first contact with psychiatry

Primary and secondary diagnoses are reported in Table 2. All individuals from the homeless group had a primary diagnosis of schizophrenia. Nineteen of the patients also fulfilled ICD-10 criteria for at least one substance use disorder (SUD) (secondary diagnosis); cannabis and alcohol were the two substances most frequently used. Seventeen patients (49%) from the homeless group had previously received a psychiatric diagnosis different from their current SSD, with pervasive developmental disorder and hyperkinetic disorder being the most common (17%). Twenty-two patients (63%) were homeless at their first contact with psychiatric services.

**Table 2:** Current and previous diagnostic characteristics and first contact with psychiatric services

	Homeless group (*n* = 35)	Domiciled group (*n* = 50)	Total sample (*N* = 85)		
Variable	*n* (%)	*n* (%)	*n* (%)	95% CI	*P*-value
Current diagnosis (ICD-10^a^)					
Schizophrenia	35 (100)	42 (84)	77 (91)	1.6	0.02
Paranoid schizophrenia	16 (46)	34 (68)	50 (59)	0.2, 1.0	0.05
Schizotypal disorder	0 (0)	7 (14)	7 (8)	0., 0.8	0.07
Hebephrenic schizophrenia	10 (29)	1 (2)	11 (13)	0, 0.4	*<0.1*
Catatonic schizophrenia	0 (0)	2 (4)	2 (2)	0, 5.0	0.3
Schizoaffective disorder, mixed type	0 (0)	1 (2)	1 (1)	0, 27.1	0.6
Simple schizophrenia	3 (9)	2 (4)	5 (6)	0.3, 19.6	0.7
Undifferentiated schizophrenia	5 (14)	2 (4)	7 (8)	0.7, 30.9	0.1
Other non-organic psychosis	0 (0)	1 (2)	1 (1)	0, 27.1	0.6
Current secondary diagnosis (ICD-10)					
Any secondary diagnosis of substance use	19 (54)	18 (36)	37 (44)	0.9, 5.1	0.1
More than one secondary diagnosis of substance use	14 (40)	9 (18)	23 (27)	1.1, 8.4	0.03
Initially allocated diagnosis (ICD-10)					
Any prior diagnosis	17 (49)	32 (64)	49 (58)	0.2, 1.5	0.3
Affective disorder	4 (11)	16 (32)	20 (24)	0.1, 0.9	0.05
Personality disorder	5 (14)	9 (18)	14 (16)	0.2, 2.5	0.9
Anxiety disorder	2 (5.7)	11 (22)	13 (15)	0, 1.0	0.08
Pervasive developmental disorder or hyperkinetic disorder	6 (17)	7 (14)	13 (15)	0.4. 4.3	>0.9
Acute psychosis	3 (8.6)	9 (18)	12 (14)	0.1, 1.7	0.4
Schizotypal disorder	3 (8.6)	8 (16)	11 (13)	0.1, 1.0	0.5
Other	4 (11)	5 (10)	9 (11)	0.3, 4.9	0.8
Unknown	4 (11)	5 (10)	9 (11)		
Type of service at first contact					
Psychiatric emergency unit	20 (57)	23 (46)	43 (51)	0.6, 3.8	0.3
HOPS/out-patient clinic^c^	6 (17)	6 (12)	12 (14)	0.4, 5.4	0.5
Child and adolescence psychiatry	6 (17)	5 (10)	11 (13)	0.5, 7.1	0.4
Psychiatric practice	1 (3)	6 (12)	7 (8)	0, 1.6	0.2
Psychotherapy clinic	1 (3)	3 (6)	4 (5)	0, 4.5	0.6
Other	1 (3)	0 (0)	1 (1)	0.1, 0	0.4
Unknown	0 (0)	7 (14)	7 (8)		
Hospitalisation					
History of psychiatric hospital admission (yes)	23 (66)	43 (86)	66 (78)	0.1, 0.9	0.03
Number of admissions, for those with an admission (mean (median) (s.d.))^b^	5.7 (2) (12.8)	6.4 (4) (7.5)	6.1 (3) (9.6)	–0.1, 0.3	0.3

a International Classification of Diseases, 10th revision (ICD-10).

b Mann–Whitney *U*-test for unequally distributed data with nonequal variance.

c Homeless Outreach Psychiatric Service (HOPS).

Bonferroni correction was applied for multiple comparisons; adjusted significance level is *α* = 0.002 (0.05/29.0). Significant *P*-values are reported in italics.

Of the domiciled group, 42 patients had a primary diagnosis of schizophrenia and 18 also fulfilled the diagnostic criteria for at least one SUD. Also in this group, the two substances most frequently used were cannabis and alcohol. Thirty-two patients (64%) in this group had previously received a diagnosis other than their current SSD, affective disorder being the most common (32%).

There were no significant differences in type of service at first contact with psychiatric services (see Table 2 for further details).

### Course of illness: onset and delays

**Fig. 1:**
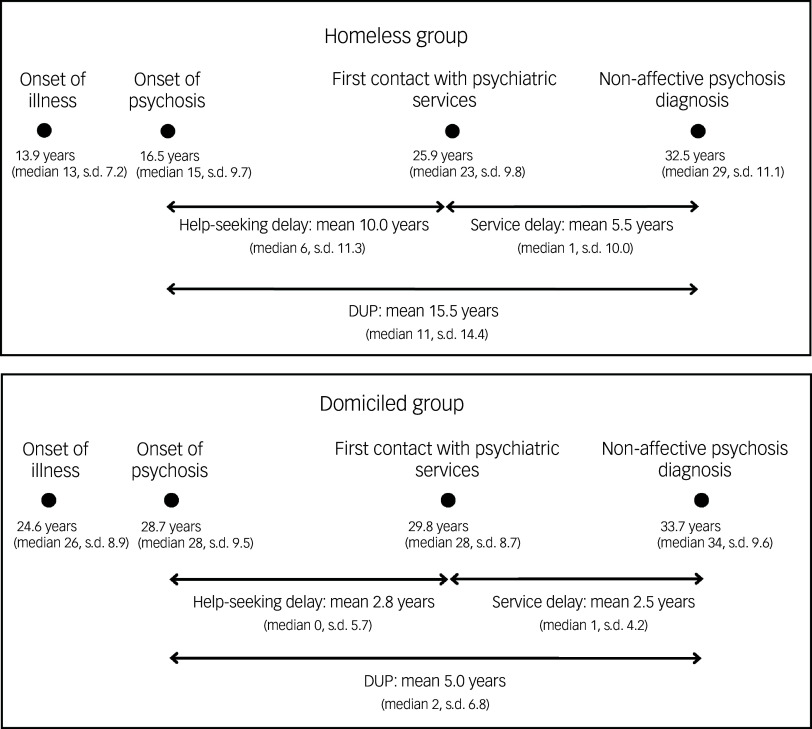
Onsets and delays. Numbers represent mean value of age, in years. Between-group differences: onset of illness, 95% CI 7.3, 14.0, *P* < 0.001; onset of psychosis, 95% CI 7.7, –17.0, *P* < 0.001; first contact with psychiatric services, 95% CI –0.4, 8.0, *P* = 0.08; age at diagnosis, 95% CI –3.0, 6.4, *P* = 0.5. DUP, duration of untreated psychosis.

Figure 1 displays the pathway to care for both groups. The homeless group showed a significantly earlier onset of illness (95% CI 7.3, 14.0, *P* < 0.001) and psychosis (95% CI 7.7, –17.0, *P* < 0.001) than the domiciled group. The two groups were of similar ages at first contact with psychiatric services (95% CI –0.4, 8.0, *P* = 0.08) and when receiving a non-affective psychosis diagnosis (95% CI –3.0, 6.4, *P* = 0.5). We found a mean service delay of 3.9 years (median 1.0, s.d. 7.5) for the whole sample; delay was longer for the homeless group (5.5 *v*. 2.5 years). In the domiciled group, service delay constituted 50% of overall delay from the onset of psychosis until diagnosis, and 35% in the homeless group.

### Course of illness: multiple regression analysis

We conducted a multiple regression analysis to examine the associations between help-seeking delay, service delay and DUP, and DUI and group, as well as other possible associated variables; the results are presented in Table 3. Group (homeless *v*. domiciled) was significantly associated with all four outcomes, in that homelessness was associated with longer delay. Older age at non-affective psychosis diagnosis was significantly associated with longer delays in all four outcomes. Migrant status predicted longer service delay and DUP. Help-seeking delay was significantly associated with increased service delay. Finally, the location of first contact was associated with service delay, in that first contact with child and adolescent psychiatric services (CAPS) was associated with longer delays (CAPS: mean service delay 12.38 (median 4.5, s.d. 16.9)). For a detailed summary of the analyses, see also supplementary material Appendix 1 available at https://doi.org/10.1192/bjo.2025.19, as well as DAG data in supplementary material Appendix 2.

**Table 3:** Multiple regression analysis for help-seeking delay, service delay, duration of untreated psychosis (DUP) and duration of untreated illness (DUI)

Variable	Help-seeking delay		Service delay		DUP		DUI	
	Beta	*P*-value	Beta	*P*-value	Beta	*P*-value	Beta	*P*-value
Group (homeless (0), domiciled (1))	–0.411	*0.009*	–2.296	*0.001*	–0.426	*<0.001*	–0.484	*<0.001*
Sex (male (0), female (1))	0.001	0.991	–0.210	0.673	–0.031	0.771	–0.053	0.575
Location of first contact	–0.001	0.992	0.230	*0.043*	0.148	0.145	0.143	0.122
Age at diagnosis	0.276	*0.033*	0.503	*<0.001*	0.487	*<0.001*	0.623	*<0.001*
Help-seeking delay	–	–	–0.309	*0.013*				
Substance use, current (no (0), yes (1))	–0.028	0.842	–0.016	0.893	–0.014	0.896	0.032	0.745
Education level	–0.056	0.651	0.189	0.082	0.080	0.409	0.123	0.157
Migrant status (no (0), yes (1))	0.213	0.113	0.294	*0.015*	0.312	*0.004*	–0.027	0.774
Prior diagnosis of anxiety or affective disorder (no (0), yes (1))	–0.169	0.189	0.094	0.405	–0.037	0.714	0.071	0.416
Prior neurodevelopmental disorder (no (0), yes (1))	–0.093	0.499	0.210	0.082	0.087	0.418	0.014	0.884
Prior schizotypal disorder (no (0), yes (1))	0.118	0.394	0.099	0.410	0.114	0.292	0.163	0.078
*R* ^2^	0.339		0.519		0.590		0.632	

Significant *P*-values are reported in italics (*P* < 0.05).

### Opposition and overcompliance

Table 4 shows the included items and group differences. There was a significant difference between the homeless and domiciled groups in total scores for opposition and overcompliance. Cronbach’s α for overcompliance was high, at 0.76, while for opposition it was moderate at 0.61.

**Table 4:** Exaggerated opposition *v*. overly compliant

Compliance, items	Homeless group, *n* = 35	Domiciled group, *n* = 50	*P*-value
Secondary ruminations	9 (26%)	14 (28%)	0.838
General anxiety	0 (0%)	9 (18%)	0.011
Social anxiety	13 (37%)	28 (56%)	0.206
Diffuse anxiety	0 (0%)	8 (16%)	0.017
Exaggerated self-blame	0 (0%)	9 (18%)	0.012
Contact affected by anxiety or insecurity	3 (9%)	13 (26%)	0.087
Overcompliance	0 (0%)	10 (20%)	0.066
Anxiety (PANSS)	14 (40%)	39 (78%)	0.008
Guilt feelings (PANSS)	3 (9%)	21 (42%)	0.024
**Total (mean score)**	**4.7 (13.4%)**	**16.8 (33.6%)**	***<0.001***
		Cronbach’s *α* 0.76	
Opposition, items	Homeless group, *n* = 35	Domiciled group, *n* = 50	*P*-value
Hostility	27 (77%)	19 (38%)	0.013
Paranoid anxiety	9 (26%)	13 (26%)	1.000
‘As if’ experience of extraordinary insight or abilities	13 (37%)	13 (26%)	0.354
Solipsistic grandiosity	21 (60%)	19 (38%)	0.137
Suspicious and guarded or marked by hostility	13 (37%)	5 (10%)	*0.007*
Aloof	6 (17%)	8 (16%)	0.898
Poor cooperation	4 (11%)	3 (6%)	0.389
Uncooperativeness (PANSS)	7 (20%)	6 (12%)	0.350
Poor impulse control (PANSS)	8 (23%)	12 (24%)	0.912
(Grossly) disorganised behaviour	9 (33%)	13 (26%)	0.988
**Total (mean score)**	**11.7 (33.4%)**	**11.1 (22.2%)**	***0.001***
		Cronbach’s *α* 0.61	

PANSS, positive and negative syndrome scale.

Two-sample *t*-test; two-sample test for equality of proportions. Bonferroni correction was applied for multiple comparisons. Adjusted significance level is *α* = 0.002 (0.05/21.0). Significant *P*-values are reported in italics.

## Discussion

### Main findings

A mean service delay of 5.5 years (287 weeks) for the homeless group and 2.5 years (130 weeks) for the domiciled group, from first contact with psychiatric services to receiving a diagnosis of schizophrenia or other non-affective psychosis, is a worrying finding in this sample of 85 patients. Our results are in line with previous findings of psychiatric services being a major barrier to the early detection of psychosis, finding a mean delay among first-episode psychosis individuals of 41.3 weeks^12^ and 18.8 weeks,^11^ respectively. However, our mean delays are radically longer than those reported in previous studies.

It merits attention that the vast majority of our sample was diagnosed within the past 10 years, i.e. long after the focus on early intervention commenced and was implemented nationwide in Denmark (OPUS).^34^

### Service delay

The prolonged service delay is a major cause for concern. However, it also holds the potential for a marked shortening of DUP because service delay contributed to 50% of DUP in the domiciled group and 35% in the homeless group. Improving detection of psychosis when and where patients first seek help in the mental health system may hold a key for marked reduction in DUP for some of the most vulnerable groups known to have long DUP, as well as for other risk factors related to severe illness progression. The pressing question is, of course, why it takes so much time to correctly identify, diagnose and treat some of the patients most impaired by schizophrenia.

In our view, several important issues may be at play here. First, it is widely recognised that first-episode psychosis can manifest in atypical ways. For instance, Kvig and colleagues^12^ discuss the ‘lanthanic presentation’, in which patients seek help for a reason unrelated to psychosis, such as anxiety or depressive symptoms.^12,35^ Such a presentation is common,^36^ also in our sample. It may stem from various factors: the illness may, in fact, initially manifest in a manner resembling other mental disorders such as anxiety or depression; the patient may be troubled more by non-psychotic symptoms, which are then subsequently reported during the initial psychiatric evaluation; or the patient may find that non-psychotic symptoms are easier to convey during this evaluation, or they may dissimulate.^37^

In an accelerated healthcare system there is a risk of diagnostic tunnel vision in which the diagnostic process focuses inadvertently on the patient’s presenting complaint, potentially overlooking, downplaying or explaining away descriptions of psychotic symptoms.^38^ This can lead to missed opportunities for proper diagnostic evaluation and treatment. For example, one study found that, among first-contact patients who reported at least one psychotic symptom, 33% were given a diagnosis unrelated to psychosis.^39^

A second concern is the incomplete, ambiguous or chaotic presentation of some clinical pictures, which can disrupt the diagnostic process or lead to misdiagnosis.^11^ This issue may be particularly pertinent for homeless patients who, as part of their illness, may oppose the diagnostic process and be mistakenly perceived by healthcare professionals as simulating symptoms.^37^ Furthermore, patients may suffer from disorganised or simple schizophrenia – diagnoses that are rarely used today and, thus, knowledge of their clinical manifestations is fading.^40^

In early diagnostic assessments, it is crucial to have sufficient time to conduct a proper assessment, to have a nuanced understanding of psychopathology and to employ an interviewing approach that can effectively reveal psychopathology.^41^ This requires a continuous prioritisation of training and supervision of doctors in psychopathology, diagnostic assessment and psychiatric interviews in a clinical setting. A more comprehensive understanding of psychopathology may help identify and initiate timely care for some of the most vulnerable patients in society today.

Such a comprehensive approach could improve access to, and the capability of, diagnostic assessment for patients seeking help for their mental health issues wherever they seek this help, e.g. at psychiatric emergency units,^42^ child and adolescent psychiatry,^43^ primary care physicians,^44^ schools,^45^ homeless shelters, substance use clinics or unemployment services.^46^

Third, we must consider the setting in which the diagnostic assessment is to take place. For severely ill patients an out-patient diagnostic process may prove difficult, if not impossible, due to discontinuation, self-isolation, lack of a social network that can assist the patient in showing up for appointments, and impaired insight into illness – all of these are well-known features of severe mental illness. For some patients, making a comprehensive diagnostic assessment and initiating appropriate treatment is possible only during hospitalisation, and it may be a way to shorten the service delay for patients with severe functioning impairment.

One of the main challenges in early-intervention and clinical high-risk (CHR) services is that many individuals who need help do not seek it. Increasing evidence suggests that early-detection programmes may not effectively identify many people who are at risk of, or who already have developed, psychosis. Studies by Ajnakina et al,^47^ Burke et al^48^ and Davies et al^49^ indicate that early-detection programmes capture only a fraction of future psychosis cases, even in areas with easily accessible and well-known CHR clinics.

### DUP and course of illness

We also found a worryingly long DUP in both groups, particularly in the homeless group (15.5 years), which we have reported elsewhere (Handest et al, submitted; details available from the author on request). The domiciled group had a mean DUP of 261 weeks (5.0 years), which markedly exceeds that for patients with schizophrenia found in other studies; for example, one meta-analysis found a mean DUP of 61.3 weeks for individuals diagnosed with broadly defined schizophrenia (the authors included studies with at least 75% diagnosed with schizophrenia or schizoaffective, schizophreniform or delusional disorder).^50^

Additionally, we identified other important differences between the two groups of patients. Compared with domiciled patients, the homeless group experienced a longer help-seeking delay, i.e. timespan from first psychotic symptom until first contact with psychiatric services. Studies have found that persons in the individual’s immediate surroundings often play a pivotal role in initiating first contact.^51^ Individuals who lack contact with close relatives, perhaps due to illness, or those whose family members are ill prepared to assist in seeking help, face the risk of prolonged delays. Various additional factors, including substance use disorder, chaotic or hostile behaviour and homelessness, can further complicate and delay help-seeking.

Furthermore, we found the homeless group to be significantly younger at onset compared with the domiciled group. This underlines early onset as a well-known prognostic factor. In a study by Tsuji and colleagues,^45^ it was found that teacher-rated premorbid social functioning was a strong predictor for a diagnosis within the schizophrenia spectrum. Thus, social functioning may be a marker of vulnerability, potentially exacerbating the risk of severe illness or, indeed, be the first sign of severe illness.

### Opposition, overcompliance and timely diagnosis and treatment

Confirming our impression during data collection, the homeless group had significantly more symptoms and signs of opposition compared with the domiciled group which, by contrast, showed significantly more symptoms and signs of overcompliance. For Binswanger, both opposition and overcompliance may be an expression of schizophrenic autism.^27^ According to Binswanger – and illustrated in his detailed case studies – patients with schizophrenia, in quite different ways, may become entrapped in their own subjective world and, to some extent, disconnected from the world we share. Opposition and overcompliance may be ways of dealing with and navigating a social world, which one has difficulties understanding and does not feel entirely at home in.^28^ In our view, these two ways of interacting with others may, although in different ways, be counterproductive in relation to seeking and receiving help, e.g. by taking an oppositional stance to others or by being so overly compliant that their illness risks being overlooked or misdiagnosed partly through their attempt to fit in, please the interviewer and do things correctly (e.g. not reporting symptoms that the interviewer had not asked about).

Our results show alarming help-seeking and service delay in two groups of patients with SSD and severe social impairment. To address these issues, we suggest a critical assessment of current diagnostic practices and of how they may affect help-seeking and service delays for vulnerable groups. This could entail mapping where and how patients seek help to direct resources and expertise to critical points of contact, as well as exploring ways to identify and assist those unable to seek help. Furthermore, we suggest a crucial prioritisation of equipping clinical staff with the skills and knowledge, enabling better and earlier recognition of the ways in which schizophrenia may manifest. To achieve this goal, some of the following initiatives could be considered: reviving teaching, training and supervision in psychopathology; prioritising comprehensive diagnostic evaluations, also in the initial phases, resisting premature referrals to specialised units based on preliminary diagnostic assessments; and improving options within psychiatry for flexibility, outreach, follow-up and evaluation during admission.

### Strengths and limitations

The study’s main strength is the comprehensive data collection through multiple face-to-face interviews, using a semi-structured interview style and adjuvanted by medical records. The limitation of a small sample size is evident, emphasising the time-consuming nature of the data collection process. Our study was conducted on two selected groups – homeless and domiciled participants with schizophrenia spectrum disorders – from two mental health services in urban areas, which limits the generalizability of our findings. Nevertheless, the study casts new light on the selected groups.

Another limitation arises from the overweight representation of female patients in the domiciled sample, and the converse of male patients in the homeless sample, as well as from the age difference between the groups, which could have skewed the data. To address this concern, we have taken precautions by adjusting for age and sex in the analyses. Our definition of DUP varies from that of Larsen et al^51^ and Birchwood et al,^7^ who recommend setting the end of DUP to the commencement of adequate antipsychotic treatment, which requires adherence or more than 1 month of continued usage, or symptom reduction. These requirements would have been challenging for some of our participants, resulting in a longer DUP. Some participants had received sporadic antipsychotic medication prior to receiving a diagnosis of schizophrenia or other non-affective psychosis, e.g. due to acute psychosis; however, none had received treatment according to the guidelines mentioned above.

Information on first contact, prior clinical evaluations and diagnoses are derived from medical records and participants’ recollections. Drawing on patients’ recollections is prone to recall bias and, thus, represents a study limitation. Recall bias may have led to some inaccuracies in the collected data, and this limitation should be considered when weighing up the study’s conclusions. For example, the onset of psychosis is a difficult time point to establish. For some participants, the time of their first psychotic symptoms could not be established or recalled and, in these cases, we noted the onset of psychosis as the first description of psychotic symptoms in the medical records. In these cases, our conservative approach may have affected DUP and the length of service delay, possibly rating them shorter than they were.

## Supporting information

Mølstrøm et al. supplementary material 1 Mølstrøm et al. supplementary material

Mølstrøm et al. supplementary material 2 Mølstrøm et al. supplementary material

## Data Availability

Data were collected and managed under strict adherence to ethical guidelines and data protection regulations. Access to the raw data is therefore restricted to ensure the privacy of the individuals involved, and to comply with applicable data protection laws such as the General Data Protection Regulation and national data protection standards.

## Supplementary material

The supplementary material for this article can be found at https://doi.org/10.1192/bjo.2025.19
