# Supplementary material for: Service delay in schizophrenia: case–control study of pathways to care among homeless and non-homeless patients
Source: BJPsych Open. 2025 Mar 25;11(2):e65. doi: 10.1192/bjo.2025.19 (PMC12001915; doi:10.1192/bjo.2025.19)
Supplement: Mølstrøm et al. supplementary material 1 — Mølstrøm et al. supplementary material [file S2056472425000195sup001.pdf]

Regression

Variables Entered/Removed[a]

Model	Variables Entered	Variables Removed	Method
1	Schizotypal disorder, Age at psychosis diagnosis, Neurodevelopmental, Help-seeking delay, Substance use, any, Education1, Køn, Where (PAM=1, GPT=2, BUP=3, PP=4, Psychotherapy=2, Outpatient=2), NEUROTIC, Migrantstatus, Group[b]	.	Enter

a. Dependent Variable: Service delay

b. All requested variables entered.

Model Summary

Model	R	R Square	Adjusted R Square	Std. Error of the Estimate
1	,720[a]	,519	,415	6,114

a. Predictors: (Constant), Schizotypal disorder, Age at psychosis diagnosis, Neurodevelopmental, Help-seeking delay, Substance use, any, Education1, Køn, Where (PAM=1, GPT=2, BUP=3, PP=4, Psychotherapy=2, Outpatient=2), NEUROTIC, Migrantstatus, Group

ANOVA[a]

Model		Sum of Squares	df	Mean Square	F	Sig.
1	Regression	2057,954	11	187,087	5,005	<,001[b]
	Residual	1906,364	51	37,380		
	Total	3964,317	62			

a. Dependent Variable: Service delay

b. Predictors: (Constant), Schizotypal disorder, Age at psychosis diagnosis, Neurodevelopmental, Help-seeking delay, Substance use, any, Education1, Køn, Where (PAM=1, GPT=2, BUP=3, PP=4, Psychotherapy=2, Outpatient=2), NEUROTIC, Migrantstatus, Group

Coefficients[a]

Model		Unstandardized Coefficients B	Unstandardized Coefficients Std. Error	Standardized Coefficients Beta	t
1	(Constant)	-13,632	4,185		-3,257
	Group	-4,696	2,198	-,296	-2,137
	Køn	-,821	1,935	-,049	-,424
	Where (PAM=1, GPT=2, BUP=3, PP=4, Psychotherapy=2, Outpatient=2)	1,879	,907	,230	2,071
	Age at psychosis diagnosis	,390	,088	,503	4,434
	Help-seeking delay	-,263	,102	-,309	-2,588
	Substance use, any	-,263	1,949	-,016	-,135
	Education1	2,262	1,277	,189	1,772
	Migrantstatus	5,937	2,352	,294	2,524
	NEUROTIC	1,673	1,992	,094	,840
	Neurodevelopmental	5,292	2,981	,210	1,775
	Schizotypal disorder	2,146	2,585	,099	,830

Coefficients[a]

Model		Sig.	95,0% Confidence Interval for B Lower Bound	95,0% Confidence Interval for B Upper Bound
1	(Constant)	,002	-22,033	-5,230
	Group	,037	-9,109	-,284
	Køn	,673	-4,706	3,064
	Where (PAM=1, GPT=2, BUP=3, PP=4, Psychotherapy=2, Outpatient=2)	,043	,058	3,701
	Age at psychosis diagnosis	<,001	,214	,567
	Help-seeking delay	,013	-,467	-,059
	Substance use, any	,893	-4,176	3,650
	Education1	,082	-,301	4,825
	Migrantstatus	,015	1,215	10,658
	NEUROTIC	,405	-2,326	5,672
	Neurodevelopmental	,082	-,693	11,277
	Schizotypal disorder	,410	-3,044	7,337

a. Dependent Variable: Service delay

Regression

Variables Entered/Removed[a]

Model	Variables Entered	Variables Removed	Method
1	Schizotypal disorder, Age at psychosis diagnosis, Neurodevelopmental, Migrantstatus, Substance use, any, Education1, NEUROTIC, Køn, Where (PAM=1, GPT=2, BUP=3, PP=4, Psychotherapy=2, Outpatient=2), Group[b]	.	Enter

a. Dependent Variable: Help-seeking delay

b. All requested variables entered.

Model Summary

Model	R	R Square	Adjusted R Square	Std. Error of the Estimate
1	,582[a]	,339	,212	8,337

a. Predictors: (Constant), Schizotypal disorder, Age at psychosis diagnosis, Neurodevelopmental, Migrantstatus, Substance use, any, Education1, NEUROTIC, Køn, Where (PAM=1, GPT=2, BUP=3, PP=4, Psychotherapy=2, Outpatient=2), ...

ANOVA[a]

Model		Sum of Squares	df	Mean Square	F	Sig.
1	Regression	1852,640	10	185,264	2,666	,010[b]
	Residual	3614,218	52	69,504		
	Total	5466,857	62			

a. Dependent Variable: Help-seeking delay

b. Predictors: (Constant), Schizotypal disorder, Age at psychosis diagnosis, Neurodevelopmental, Migrantstatus, Substance use, any, Education1, NEUROTIC, Køn, Where (PAM=1, GPT=2, BUP=3, PP=4, Psychotherapy=2, Outpatient=2), Group

Coefficients[a]

Model		Unstandardized Coefficients B	Unstandardized Coefficients Std. Error	Standardized Coefficients Beta	t
1	(Constant)	2,775	5,693		,487
	Group	-7,659	2,802	-,411	-2,733
	Køn	,028	2,639	,001	,011
	Where (PAM=1, GPT=2, BUP=3, PP=4, Psychotherapy=2, Outpatient=2)	-,013	1,237	-,001	-,011
	Age at psychosis diagnosis	,251	,115	,276	2,188
	Substance use, any	-,533	2,657	-,028	-,201
	Education1	-,791	1,737	-,056	-,456
	Migrantstatus	5,042	3,130	,213	1,611
	NEUROTIC	-3,555	2,671	-,169	-1,331
	Neurodevelopmental	-2,759	4,047	-,093	-,682
	Schizotypal disorder	3,011	3,501	,118	,860

Coefficients[a]

Model		Sig.	95,0% Confidence Interval for B Lower Bound	95,0% Confidence Interval for B Upper Bound
1	(Constant)	,628	-8,650	14,199
	Group	,009	-13,283	-2,036
	Køn	,991	-5,267	5,323
	Where (PAM=1, GPT=2, BUP=3, PP=4, Psychotherapy=2, Outpatient=2)	,992	-2,496	2,469
	Age at psychosis diagnosis	,033	,021	,482
	Substance use, any	,842	-5,865	4,798
	Education1	,651	-4,278	2,695
	Migrantstatus	,113	-1,239	11,322
	NEUROTIC	,189	-8,915	1,806
	Neurodevelopmental	,499	-10,880	5,363
	Schizotypal disorder	,394	-4,013	10,036

a. Dependent Variable: Help-seeking delay

Regression

Variables Entered/Removed[a]

Model	Variables Entered	Variables Removed	Method
1	Schizotypal disorder, Age at psychosis diagnosis, Neurodevelopmental, Migrantstatus, Substance use, any, Education1, NEUROTIC, Køn, Where (PAM=1, GPT=2, BUP=3, PP=4, Psychotherapy=2, Outpatient=2), Group[b]	.	Enter

a. Dependent Variable: DUP

b. All requested variables entered.

Model Summary

Model	R	R Square	Adjusted R Square	Std. Error of the Estimate
1	,768[a]	,590	,513	8,584

a. Predictors: (Constant), Schizotypal disorder, Age at psychosis diagnosis, Neurodevelopmental, Migrantstatus, Substance use, any, Education1, NEUROTIC, Køn, Where (PAM=1, GPT=2, BUP=3, PP=4, Psychotherapy=2, Outpatient=2), ...

ANOVA[a]

Model		Sum of Squares	df	Mean Square	F	Sig.
1	Regression	5625,944	10	562,594	7,635	<,001[b]
	Residual	3905,493	53	73,689		
	Total	9531,438	63			

a. Dependent Variable: DUP

b. Predictors: (Constant), Schizotypal disorder, Age at psychosis diagnosis, Neurodevelopmental, Migrantstatus, Substance use, any, Education1, NEUROTIC, Køn, Where (PAM=1, GPT=2, BUP=3, PP=4, Psychotherapy=2, Outpatient=2), Group

Coefficients[a]

Model		Unstandardized Coefficients B	Std. Error	Standardized Coefficients Beta	t
1	(Constant)	-11,809	5,862		-2,015
	Group	-10,410	2,863	-,426	-3,636
	Køn	-,788	2,690	-,031	-,293
	Where (PAM=1, GPT=2, BUP=3, PP=4, Psychotherapy=2, Outpatient=2)	1,881	1,271	,148	1,480
	Age at psychosis diagnosis	,585	,118	,487	4,953
	Substance use, any	-,358	2,733	-,014	-,131
	Education1	1,484	1,782	,080	,833
	Migrantstatus	9,743	3,222	,312	3,023
	NEUROTIC	-1,011	2,744	-,037	-,369
	Neurodevelopmental	3,390	4,157	,087	,815
	Schizotypal disorder	3,835	3,602	,114	1,065

Coefficients[a]

Model		Sig.	95,0% Confidence Interval for B Lower Bound	Upper Bound
1	(Constant)	,049	-23,566	-,052
	Group	<,001	-16,154	-4,667
	Køn	,771	-6,182	4,607
	Where (PAM=1, GPT=2, BUP=3, PP=4, Psychotherapy=2, Outpatient=2)	,145	-,668	4,431
	Age at psychosis diagnosis	<,001	,348	,822
	Substance use, any	,896	-5,839	5,124
	Education1	,409	-2,090	5,058
	Migrantstatus	,004	3,279	16,206
	NEUROTIC	,714	-6,516	4,493
	Neurodevelopmental	,418	-4,949	11,728
	Schizotypal disorder	,292	-3,391	11,061

a. Dependent Variable: DUP

Regression

Variables Entered/Removed[a]

Model	Variables Entered	Variables Removed	Method
1	Schizotypal disorder, Education1, Migrantstatus, Substance use, any, Køn, Neurodevelopmental, NEUROTIC, Age at psychosis diagnosis, Where (PAM=1, GPT=2, BUP=3, PP=4, Psychotherapy =2, Outpatient=2), ...[b]	.	Enter

a. Dependent Variable: DUI

b. All requested variables entered.

Model Summary

Model	R	R Square	Adjusted R Square	Std. Error of the Estimate
1	,795[a]	,632	,569	7,651

a. Predictors: (Constant), Schizotypal disorder, Education1, Migrantstatus, Substance use, any, Køn, Neurodevelopmental, NEUROTIC, Age at psychosis diagnosis, Where (PAM=1, GPT=2, BUP=3, PP=4, ...

ANOVA[a]

Model		Sum of Squares	df	Mean Square	F	Sig.
1	Regression	5842,565	10	584,256	9,981	<,001[b]
	Residual	3395,174	58	58,537		
	Total	9237,739	68			

a. Dependent Variable: DUI

b. Predictors: (Constant), Schizotypal disorder, Education1, Migrantstatus, Substance use, any, Køn, Neurodevelopmental, NEUROTIC, Age at psychosis diagnosis, Where (PAM=1, GPT=2, BUP=3, PP=4, Psychotherapy=2, Outpatient=2), Group

Coefficients[a]

Model		Unstandardized Coefficients B	Unstandardized Coefficients Std. Error	Standardized Coefficients Beta	t
1	(Constant)	-11,651	5,061		-2,302
	Group	-11,190	2,510	-,484	-4,457
	Køn	-1,300	2,308	-,053	-,563
	Where (PAM=1, GPT=2, BUP=3, PP=4, Psychotherapy=2, Outpatient=2)	1,693	1,079	,143	1,570
	Age at psychosis diagnosis	,717	,102	,623	7,029
	Substance use, any	,774	2,369	,032	,327
	Education1	2,121	1,479	,123	1,434
	Migrantstatus	-,788	2,735	-,027	-,288
	NEUROTIC	1,851	2,262	,071	,818
	Neurodevelopmental	,538	3,659	,014	,147
	Schizotypal disorder	5,138	2,862	,163	1,795

Coefficients[a]

Model		Sig.	95,0% Confidence Interval for B Lower Bound	95,0% Confidence Interval for B Upper Bound
1	(Constant)	,025	-21,782	-1,519
	Group	<,001	-16,215	-6,165
	Køn	,575	-5,919	3,320
	Where (PAM=1, GPT=2, BUP=3, PP=4, Psychotherapy=2, Outpatient=2)	,122	-,466	3,853
	Age at psychosis diagnosis	<,001	,513	,922
	Substance use, any	,745	-3,969	5,516
	Education1	,157	-,840	5,082
	Migrantstatus	,774	-6,263	4,687
	NEUROTIC	,416	-2,676	6,378
	Neurodevelopmental	,884	-6,787	7,863
	Schizotypal disorder	,078	-,591	10,867

a. Dependent Variable: DUI
