# Supplementary material for: Service delay in schizophrenia: case–control study of pathways to care among homeless and non-homeless patients
Source: BJPsych Open. 2025 Mar 25;11(2):e65. doi: 10.1192/bjo.2025.19 (PMC12001915; doi:10.1192/bjo.2025.19)
Supplement: Mølstrøm et al. supplementary material 2 — Mølstrøm et al. supplementary material [file S2056472425000195sup002.docx]

**Appendix 2:** Directed Acyclic Diagram (DAG) for help-seeking delay, service delay, DUP and DUI


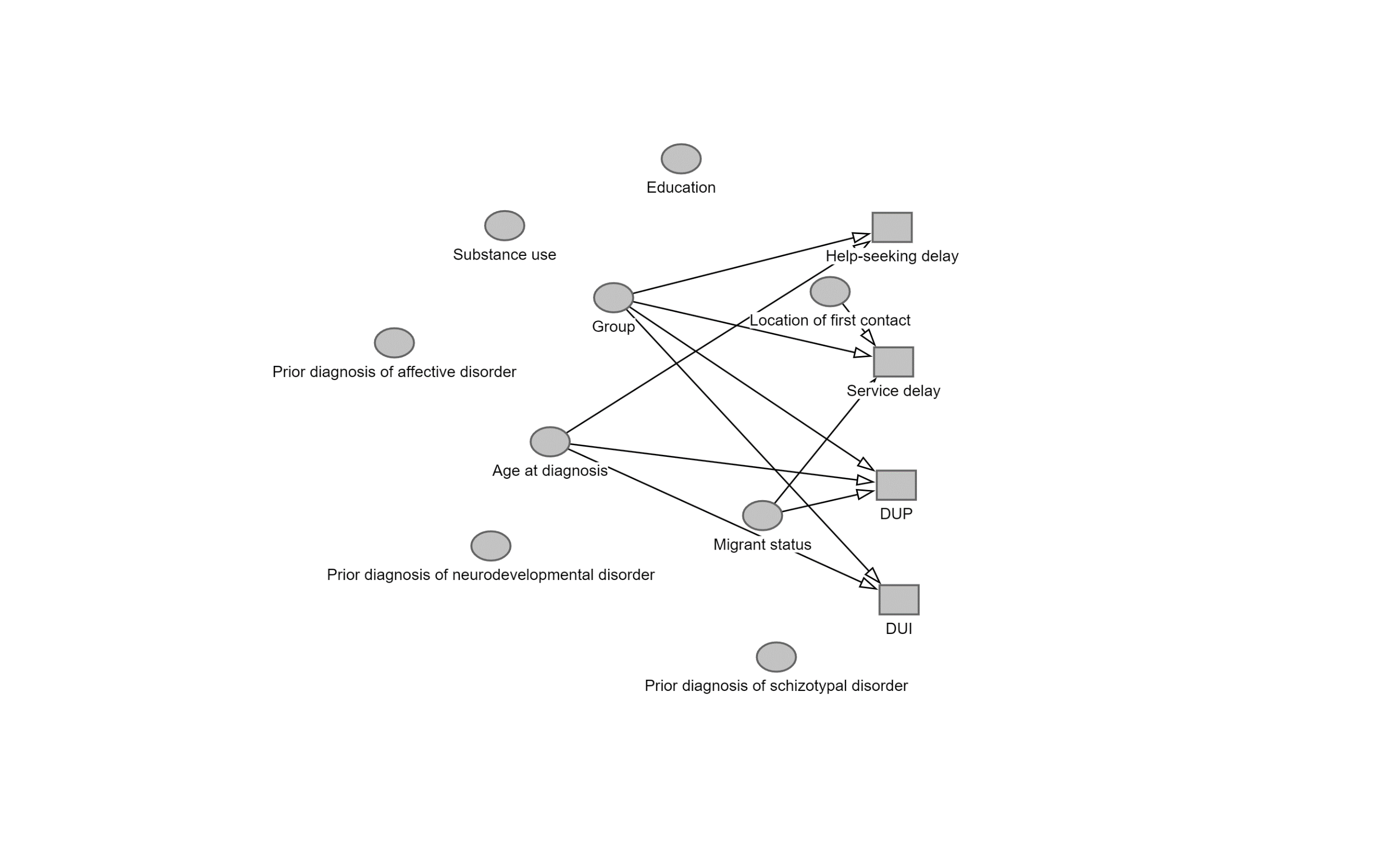


The DAG displays the associations between help-seeking delay, service delay, duration of untreated psychosis (DUP), duration of untreated illness (DUI), and Group (homeless vs domiciled), sex (male/female), location of first contact, age at non-affective psychosis diagnosis, substance use disorder, education level, migrant status, and prior diagnosis of anxiety or affective disorder, neurodevelopmental disorder, or schizotypal disorder. Arrows indicates statistically significant association (p<0.05).
